# Long non‐coding RNA ESCCAL‐1/miR‐590/LRP6 signaling pathway participates in the progression of esophageal squamous cell carcinoma

**DOI:** 10.1002/cam4.4915

**Published:** 2022-06-02

**Authors:** Hongya Guan, Pengju Lv, Pengli Han, Lijuan Zhou, Jia Liu, Wei Wu, Ming Yan, Qinghe Xing, Wei Cao

**Affiliations:** ^1^ Department of translational Medical Center Zhengzhou Central Hospital Affiliated to Zhengzhou University Zhengzhou China; ^2^ Department of Medicine University of California, San Francisco San Francisco CA USA; ^3^ Basic Medical College Zhengzhou University Zhengzhou China; ^4^ Institutes of Biomedical Sciences and Children's Hospital Fudan University Shanghai China; ^5^ Henan Diagnosis of Tumor Pathology Postdoctoral Workstation Zhengzhou China

**Keywords:** EMT, ESCC, ESCCAL‐1, miR‐590, LRP6

## Abstract

**Background:**

Long non‐coding RNAs (lncRNAs) have critical functions within esophageal squamous cell carcinoma (ESCC). However, the function and mechanism underlying ESCC‐associated lncRNA‐1 (ESCCAL‐1) in ESCC tumorigenesis have not been well clarified.

**Methods:**

ESCCAL‐1, miR‐590 and LRP6 were quantified using qRT‐PCR. Cell viability, migration and invasion abilities were measured using CCK‐8 assay and transwell assays. The protein pression was determined with western blot assay. The xenograft model assays were used to examine the impact of ESCCAL‐1 on tumorigenic effect in vivo. Direct relationships among ESCCAL‐1, miR‐590 and LRP6 were confirmed using dual‐luciferase reporter assays.

**Results:**

The present work discovered the ESCCAL‐1 up‐regulation within ESCC. Furthermore, ESCCAL‐1 was found to interact with miR‐590 and consequently restrict its expression. Functionally, knocking down ESCCAL‐1 or over‐expressing miR‐590 hindered ESCC cell growth, invasion, and migration in vitro. Moreover, inhibition of miR‐590 could reverse the effect of knockdown of ESCCAL‐1 on cells. Importantly, it was confirmed that LRP6 was miR‐590’s downstream target and LRP6 over‐expression also partly abolished the role of miR‐590 overexpression in ESCC cells.

**Conclusion:**

We have uncovered a novel regulatory network comprising aberrant interaction of ESCCAL‐1/miR‐590/LRP6 participated in ESCC progression.

## Graphical abstract

It was confirmed that ESCCAL‐1 was found to interact with miR‐590 and consequently restrict its expression. LRP6 was miR‐590's downstream target and LRP6 over‐expression also partly abolished the role of miR‐590 overexpression in ESCC cells. We manifested that a novel lncRNA ESCCAL‐1 promoted ESCC cell malignant behavior through a regulatory network of the ESCCAL‐1/miR‐590/LRP6 axis.

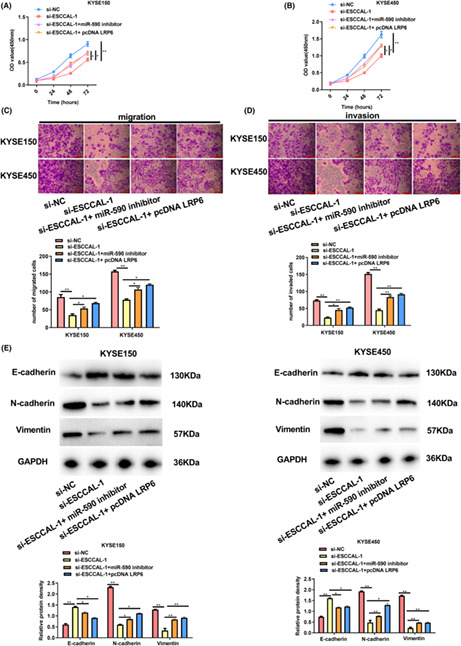

## 1 INTRODUCTION

Esophageal carcinoma (EC) ranks eighth among the frequently seen malignancies globally. EC can be classified as esophageal adenocarcinoma (EA) and ESCC histologically. ESCC represents a commonly seen EC subtype in China and the Middle East, compared to Western countries. Despite significant advances in the treatment modalities in recent years, EC still has a low 5‐year survival of <30% because of lacking effective early diagnosis and higher relapse and metastasis rates. Consequently, finding clinically valuable biomarkers is essential for early detection and effective targeted treatment for ESCC.

LncRNAs represent endogenous RNA molecules 200–100,000 nucleotides (nt) long,^1^ which participate in almost every biological and pathological process, such as cell‐cycle regulation, embryonic development, cell differentiation, and tumorigenesis.^2^, ^3^, ^4^, ^5^ As revealed by recent evidence, lncRNAs are related to EC growth and progression.^6^ The ESCC‐associated lncRNA transcript 1 (ESCCAL‐1), a novel lncRNA initially identified by our team, was up‐regulated in ESCC tumor tissues.^7^, ^8^, ^9^ Silencing ESCCAL‐1 suppressed ESCC cell invasion, enhanced apoptosis in vitro, and inhibited cancer proliferation by inactivating Src and activating the p38α pathway in the ESCC xenograft mouse model.^6^, ^10^ The potential function and possible regulatory role of ESCCAL‐1 in ESCC are not fully understood.

MiRNAs are short non‐coding RNAs (ncRNAs) about 20–25 nt long, and they are related to tumor occurrence and development.^11^ MiRNAs are widely found in many organisms, including plants, animals, and viruses. They play essential roles in a variety of tumors.^12^ MiR‐590 was reported to be a suppressor of breast cancer (BC),^13^ ESCC,^14^ non‐small cell lung cancer (NSCLC),^15^ hepatocellular carcinoma (HCC),^16^ osteosarcoma,^17^ pancreatic cancer,^18^ and colorectal cancer (CRC).^19^ Here, miR‐590 showed down‐regulation within ESCC. Even so, miR‐590's role within ESCC is not completely clarified yet.

Low‐density lipoprotein (LDL) receptor‐related protein‐6 (LRP6), a critical co‐receptor for the Wnt pathway, has been proved to involve in the tumor epithelial‐to‐mesenchymal transition (EMT) process.^11^ It was highly expressed in CRC, clear cell renal cell carcinoma (ccRCC), BC, and ESCC.^11^, ^20^, ^21^, ^22^ Emerging evidence suggests that mutual interactions among lncRNA‐miRNA‐mRNA can form regulatory networks involved in cancer.^23^ Therefore, we hypothesize that ESCCAL‐1‐miR‐590‐LRP6 interactions occur in ESCC progression.

In this study, we reported ESCCAL‐1/miR‐590/LRP6 axis in the progression of ESCC. Such ESCCAL‐1‐mediated regulatory network in ESCC may provide a new framework of lncRNA functionality in ESCC and is the potential marker used to predict ESCC prognosis and treat ESCC.

## 2 MATERIALS AND METHODS

### 2.1 Tissue specimens

Thirty paired ESCC and matched non‐carcinoma samples were provided by the Linzhou Cancer Hospital from 2017 to 2019. All cases did not receive chemotherapy or radiation preoperatively. Pathologists made the diagnosis of ESCC. Afterward, liquid nitrogen freezing and preservation of specimens under −80°C were performed before subsequent analysis. All of these subjects provided written informed consent. The Ethics Committee of Zhengzhou Central Hospital Affiliated to Zhengzhou University approved the study (approval no. 201910).

### 2.2 Cell culture

The immortal esophageal epithelial HET‐1A cells and ESCC cells (KYSE150, KYSE450) were provided by Shanghai Institutes for Biological Science (Shanghai, China). The cells were cultivated within DMEM that contained 10% fetal bovine serum (FBS, Hyclone, Logan, UT, USA), followed by incubation under 5% CO_2_ and 37°C conditions.

### 2.3 Cell transfection

GenePharma (Shanghai, China) was responsible for synthesizing ESCCAL‐1 siRNA, lentiviral short hairpin vectors overexpressing ESCCAL‐1 (si‐ESCCAL‐1), miR‐590 inhibitor, and mimic. Lipofectamine™ 2000 (Invitrogen, Carlsbad, CA, USA) was adopted in cell transfection.

### 2.4 Quantitative Real‐Time PCR (qRT‐PCR)

Total RNAs were isolated from ESCC and matched non‐carcinoma samples and ESCC cell lines by TRIzol (Invitrogen, Carlsbad, CA, USA). After that, total RNAs (1000 ng) were prepared into cDNA through reverse transcription. Gene expression was assessed by SYBR Green Master Mix (Takara, Dalian, China) with ABI 7500 system. We adopted the 2^−ΔΔCt^ method for calculating relative gene expression, which was normalized to the GAPDH expression. All primer sequences are displayed in Table 1.

**TABLE 1:** The primer sequences

Gene	Sequence(5′to 3′)
ESCCAL‐1	
Forward	CCAGACAGCAGCAAAGCAAT
Reverse	GGAAGCAGCAAATGTGTCCAT
GAPDH	
Forward	CAAGGTCATCCATGACAACTTTG
Reverse	GTCCACCACCCTGTTGCTGTAG
miR‐590	
Forward	TAATTTTATGTATAAGCTAGT
Reverse	TGGTGTCGTGGAGTCG
U6	
Forward	TCCGATCGTGAAGCGTTC
Reverse	GTGCAGGGTCCGAGGT

### 2.5 Cell counting (CCK)‐8 assay

In brief, we inoculated the transfected KYSE150 and KYSE450 cells (1 × 10^4^/well) into the 96‐well plates, and five replicates were set for each group and incubated for 24 h, 48 h, and 72 h, separately. After adding CCK‐8 solution (10 μl, Beyotime, Jiangsu, China), we incubated cells for a 4 h period under 37°C and measured absorbance (OD) values at 450 nm on the microplate reader.

### 2.6 Transwell Migration/Invasion assay

Transwell chambers (Costar, Lowell, MA, U.S.) placed in the 24‐well plates were utilized to analyze cell migration/invasion. Matrigel was coated into the top Transwell chamber ahead of time to analyze cell invasion, followed by 30‐min solidification under 37°C and 5% CO_2_ conditions. After transfection, trypsinization of KYSE150 and KYSE450 cells was conducted, followed by resuspension within a serum‐free medium, with the concentration being set as 2 × 10^6^ cells/ml. After that, 200 μl cell suspension in 500 μl DMEM that contained 10% FBS was added into the bottom chamber for a 48‐h period. Afterward, transwell chambers were subject to 10% methanol fixation and crystal violet staining. The inverted microscope was utilized to count cell numbers. Migration was analyzed by a protocol close to invasion, except for non‐Matrigel coating. Every assay was carried out thrice.

### 2.7 Luciferase reporter assay

We amplified wild‐type (ESCCAL‐1‐wt) and the mutated (ESCCAL‐1‐mut) miR‐590 binding site sequences and then inserted them in a pmirglo Dual‐luciferase miRNA Target Expression Vectors (Promega). By adopting LipofectamineTM2000, the co‐transfection of miR‐590 mimics, miR‐590 mimics NC with ESCCAL‐1‐wt, or ESCCAL‐1 mutant reporter plasmid was conducted in HEK‐293 T cells. Luciferase activities were later detected after transfection for 48 h by adopting a Dual‐Lucifer Reporter Assay System (Promega, Madison, WI, USA). Also, 3′‐UTR sequence of LRP6 that contained pmirGLO‐LRP6‐wt) or pmirGLO‐LRP6‐mut sequence of miR‐590 binding sites were cloned, transfected into cells to determine the interaction between LRP6 and miR‐590.

### 2.8 Western blotting(WB) assay

Cells in different treatment groups were rinsed by PBS, followed by 30‐min on‐ice lysis within RIPA buffer (Beyotime, Jiangsu, China) containing PMSF. Then, the BCA protein detection kit (Beyotime) was utilized to detect total protein content. The equivalent protein amount from each sample was isolated by SDS‐PAGE and subsequently transported on PVDF membranes (Millipore, Billerica, MA, USA). Afterward, 5% skim milk was adopted to block the membrane for a 2‐h period under ambient temperature, followed by subsequent incubation using the β‐catenin (1:500, Santa Cruz Biotechnology), c‐myc (1:800, Santa Cruz), cyclin D1 (1:200, Santa Cruz), E‐cadherin (1:500, Bioworld), N‐cadherin (1:500, Santa Cruz) and Vimentin (1:500, Santa Cruz) primary antibodies under 4 °C overnight. Following incubation, we rinsed membranes three times using 1 × TBST buffer, followed by incubation using a respective secondary antibody for a 2‐h period under ambient temperature. Signals were detected with ECL Plus Detection Kit (Pierce, Rockford, IL, U.S.). GAPDH served as the internal control for normalization.

### 2.9 Bioinformatic analysis

GEPIA database was to analyze ESCCAL‐1 and LRP6 expression in ESCC samples. LncBase Predicted V.2 software and TargetScan 7.2 databases were to seek binding sites of miR‐590 in ESCCAL‐1 and LRP6 3′ UTR.

### 2.10 Tumor xenograft experiments

4‐week‐old male BALB/c nude mice were purchased from Beijing Huafukang Biotechnology Co., Ltd (Beijing, China). All experimental animal procedures were performed following the Ethics Committee of Zhengzhou Central Hospital Affiliated to Zhengzhou University. All mice were placed on a 12‐hour light/dark cycle and were automatically given food and water. KYSE150 cells infected with ESCCAL_1‐shRNA or non‐sense shRNA lentivirus were used for xenotransplantation. From day 7 to day 22, tumor volume was calculated every 4 days as length × width^2^ × 0.5. Mice were sacrificed by cervical spondylosis on day 22 after injection. The transplanted tumor tissue was quickly taken out, and the tumor body was weighed. The tissues were preserved in liquid nitrogen for the next step of western blot experiments.

### 2.11 Statistical analysis

Statistical analyses were completed by SPSS21.0. All results were displayed in the form of mean ± SD. The Student's *t*‐test was used for differences between two groups, or one‐way ANOVA was used for differences between multiple groups. *P* < 0.05 stood for statistical significance.

## 3 RESULTS

### 3.1 ESCCAL‐1 silencing restrains the growth, migration, and invasion of ESCC cells

**FIGURE 1:**
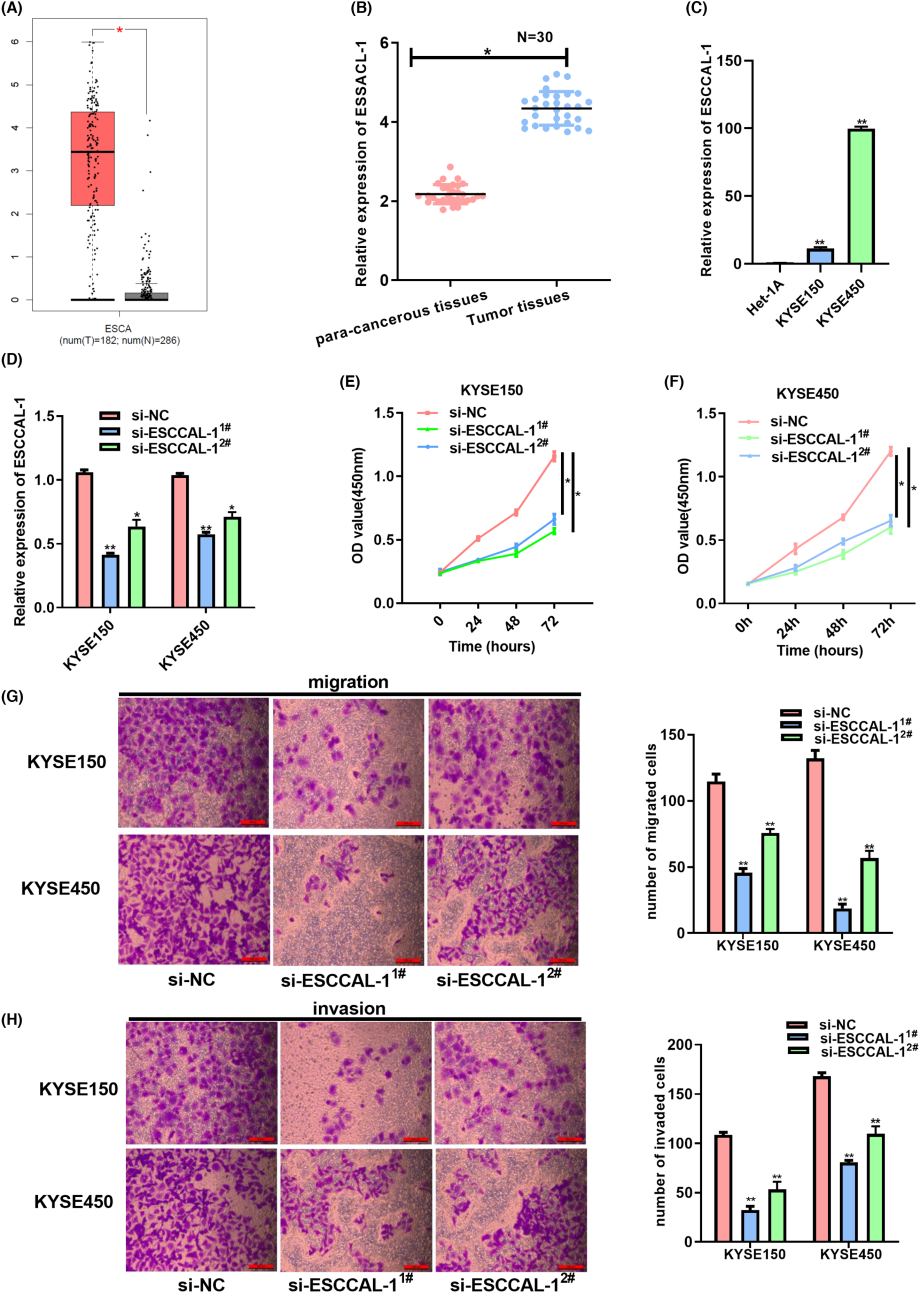
Knockdown of ESCCAL‐1 inhibits ESCC cell growth, invasion, and metastasis. (A) ESCCAL‐1 levels within ESCC tumor samples were predicted by the GEPIA website. (B) ESCCAL‐1 expression in 30 human ESCC clinical tissues was assessed by RTq‐PCR. (C) ESCCAL‐1 levels within KYSE150/KYSE450 cells were assessed through qRT‐PCR. (D) Detection of transfection efficiency ESCCAL‐1 siRNAs in KYSE150 and KYSE450 cells. (E) ESCCAL‐1 silencing's impact on KYSE150 cell proliferation was analyzed through a CCK‐8 assay. (F) ESCCAL‐1 silencing's impact on KYSE450 cell proliferation was evaluated by CCK‐8 assay. (G) ESCCAL‐1 silencing's impact on cell migration was evaluated by Transwell migration assay. (H) ESCCAL‐1 silencing's impact on cell invasion abilities was evaluated through the Transwell invasion assay. *P < 0.05, **P < 0.01.

First, the GEPIA database was used to analyze the ESCCAL‐1 level, and its expression increased within ESCC specimens relative to healthy samples (Figure 1A). We then detected ESCCAL‐1 expression in 30 paired ESCC tumors and cells. The results showed that ESCCAL‐1 expression observably increased within ESCC specimens (Figure 1B). Lymph node metastasis (LNM) and TNM stages represented 2 clinicopathologic features related to ESCCAL‐1 expression in the clinical samples (Table 2). Additionally, ESCCAL‐1 expression markedly increased in ESCC cells (Figure 1C). The two siRNAs were designed to detect the knockdown efficiency, and the highest efficiency (si‐ESCCAL‐1^1#^) was chosen for functional experiments (Figure 1D). Experiments demonstrated that silencing ESCCAL‐1 resulted in a significant decrease in growth, invasion, and migration (Figure 1E–H). Collectively, the above findings suggested oncogenic properties of ESCCAL‐1 in ESCC cells.

**TABLE 2:** Association of ESCCAL‐1 expression and clinicopathological characteristics in ESCC patients

Variables	Cases	ESCCAL‐1 expression		*P*‐value
		Low	High	
Gender				0.656
Male	20	6	14	
Female	10	3	7	
Age (years)				0.657
<60	7	2	5	
≥60	23	7	16	
Lymph node metastasis				0.018*
No	13	7	6	
Yes	17	2	15	
TNM stage				0.032*
I/II	14	7	7	
III/IV	16	2	14	
Distant metastasis				0.483
No	28	9	19	
Yes	2	0	2	

* *P* < 0.05.

### 3.2 MiR‐590 is a downstream target of ESCCAL‐1

**FIGURE 2:**
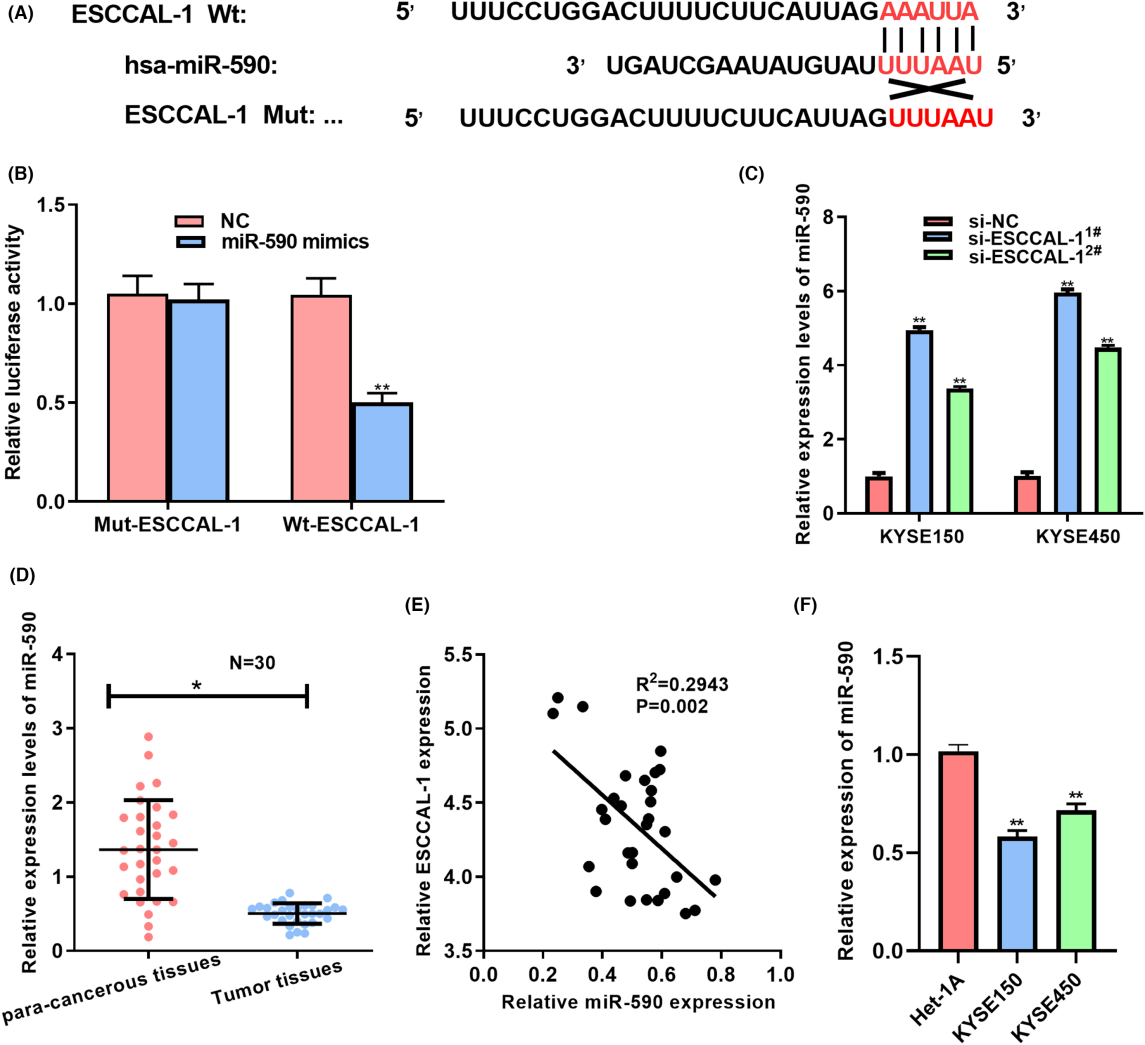
MiR‐590 serves as ESCCAL‐1's downstream target. (A) Those predicted complementary binding sites of miR‐590 and ESCCAL‐1. (B) Luciferase activities were detected within cells subject to co‐transfection of MUT or WT ESCCAL‐1 luciferase reporter plasmid with NC or miR‐590 mimics. (C) miR‐590 levels within ESCCAL‐1 knockdown KYSE150/KYSE450 cells were measured via qRT‐PCR assay. (D) miR‐590 levels among 30 human ESCC tissue samples. (E) Correlation analysis of miR‐590 expression with ESCCAL‐1 in ESCC tissues (F) miR‐590 level within KYSE150/KYSE450 cells measured through qRT‐PCR. *P < 0.05, **P < 0.01.

For elucidating the ESCCAL‐1's effect on ESCC development at the molecular level, this study utilized LncBase Predicted v.2 of the DIANA bioinformatics tool to predict potential microRNA binding sites on ESCCAL‐1 transcript. We found that miR‐590 interacted with ESCCAL‐1 (Figure 2A), as evidenced through luciferase assay (Figure 2B). Subsequently, this study attempted to see whether ESCCAL‐1 could influence the intracellular miR‐590 level. We observed that miR‐590 was increased upon down‐regulation of ESCCAL‐1 in KYSE150 and KYSE450 cells (Figure 2C). Moreover, miR‐590 presented a low expression in ESCC tissues, negatively correlated with ESCCAL‐1 transcript within ESCC samples (Figure 2D,E). MiR‐590 expression decreased within ESCC cells (Figure 2F). Collectively, ESCCAL‐1 directly binds with miR‐590 in ESCC cells.

### 3.3 miR‐590 over‐expression suppresses ESCC cell growth, invasion, and migration

**FIGURE 3:**
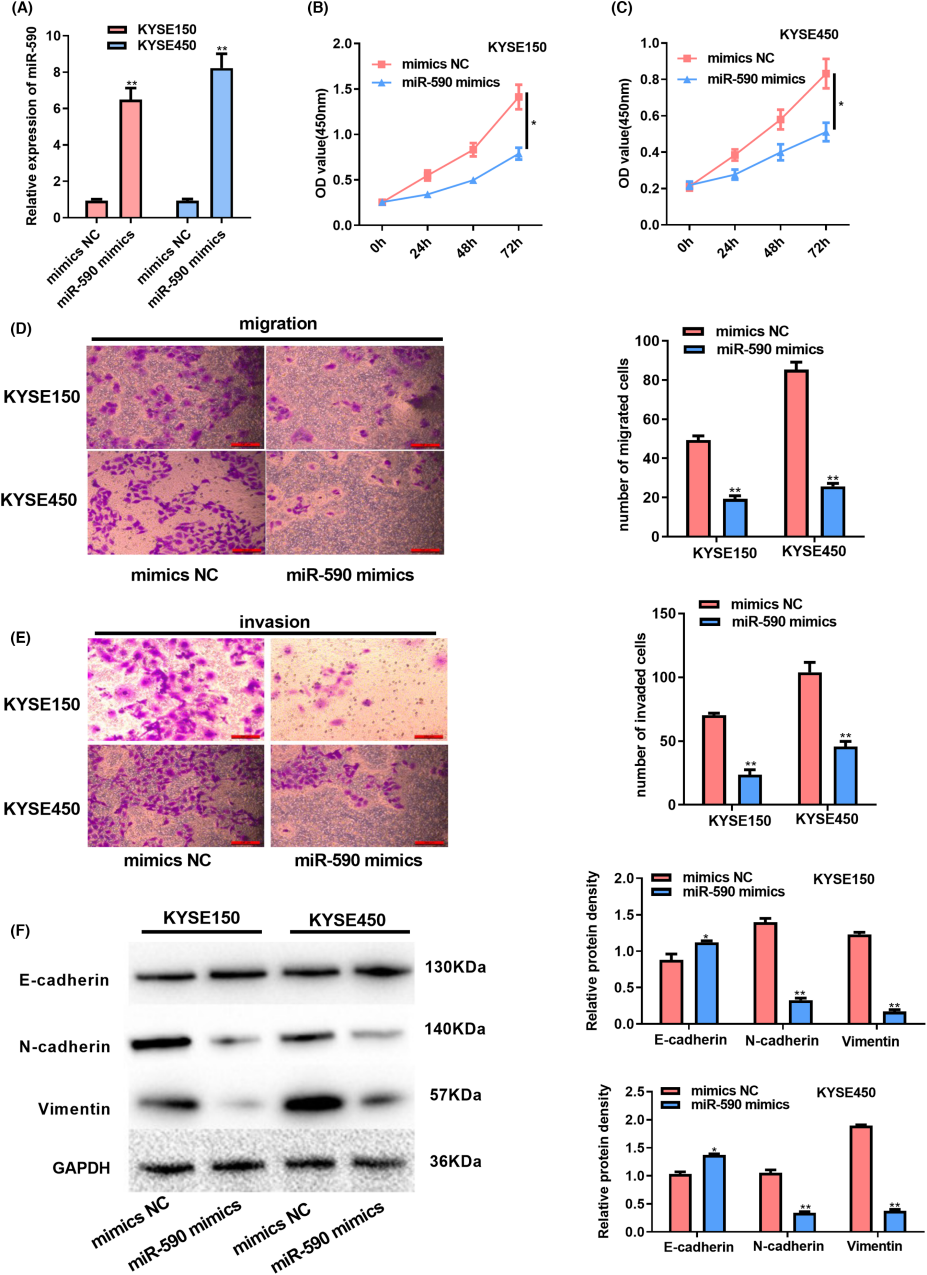
Over‐expression of miR‐590 suppresses ESCC cell proliferation, invasion, and migration. Cells were subject to miR‐590 mimic transfection. (A) Detection of transfection efficiency. (B) Cell proliferation abilities of KYSE150 cells measured by CCK‐8 assay. (C) CCK‐8 assay evaluated the growth abilities of KYSE450 cells. (D) Transwell migration assay evaluated cells' migration abilities. (E) Transwell invasion assay evaluated cell invasion abilities. (F) Western blotting assay evaluated levels of EMT‐associated proteins. *P < 0.05, **P < 0.01.

This study further explored miR‐590's function within ESCC cells. From Figure 3A, miR‐590 mimic transfection successfully up‐regulated miR‐590 within KYSE150 and KYSE450 cells. In addition, the proliferation, migratory and invasive capacity of the above two cell lines declined after the over‐expression of miR‐590 (Figure 3B–E). More importantly, over‐expression of miR‐590 could decrease N‐cadherin and Vimentin protein levels but increase the E‐cadherin level, suggesting miR‐590 acts as a tumor suppressor that restrains the EMT process in ESCC (Figure 3F). Collectively, these data revealed that increased expression of miR‐590 could restrain the ESCC phenotype.

### 3.4 Inhibiting miR‐590 reversed ESCCAL‐1 silencing's effect on ESCC cells

**FIGURE 4:**
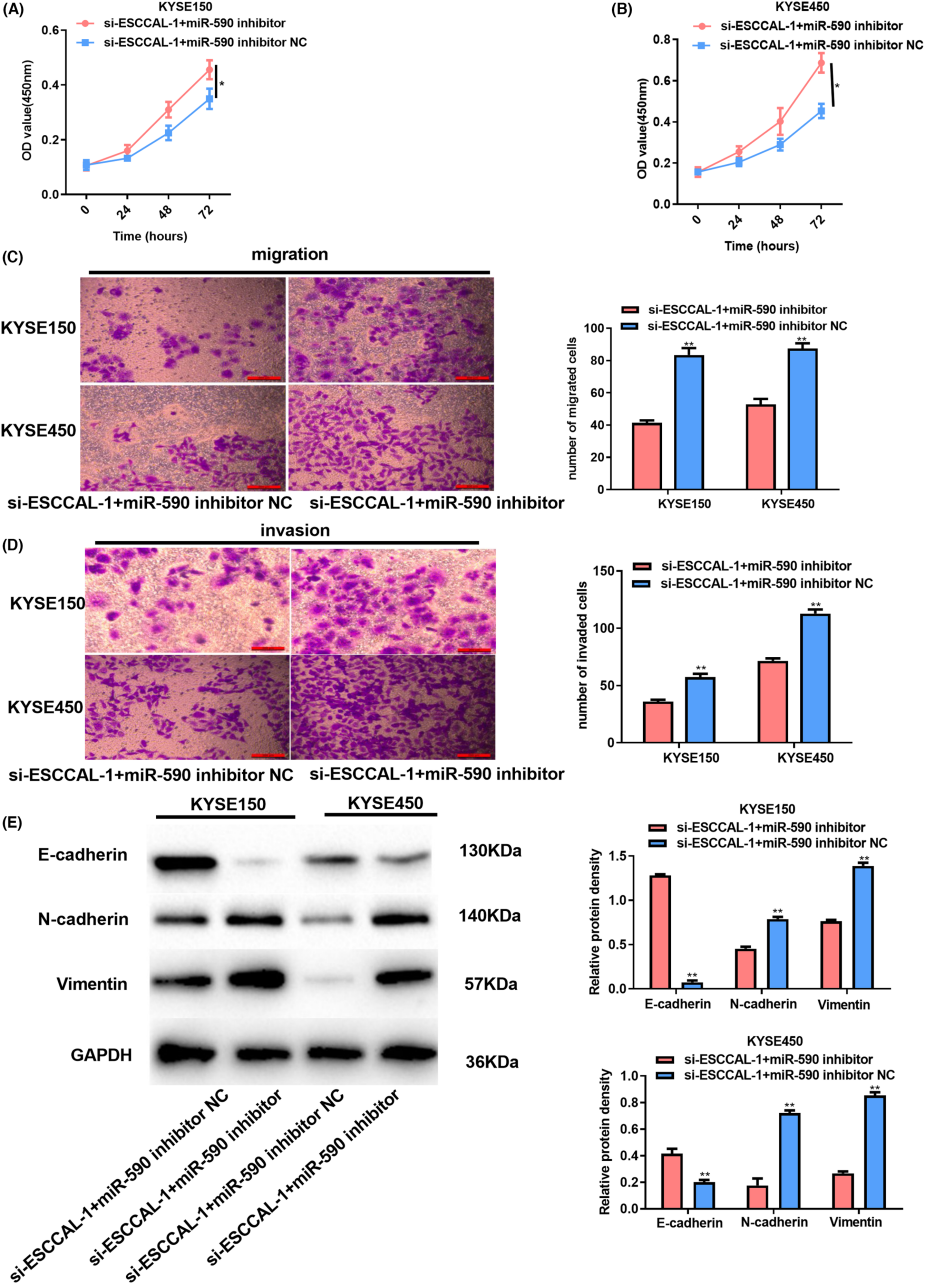
Inhibiting miR‐590 reversed the role of ESCCAL‐1 silencing in ESCC cells. Cells were co‐transfected with si‐ESCCAL‐1 with miR‐590 inhibitor. (A) CCK‐8 assay evaluated KYSE150 cell growth. (B) CCK‐8 assay evaluated KYSE450 cell growth. (C) Cell migration abilities were evaluated by Transwell migration assay. (D) Cell invasion was analyzed via Transwell invasion assay. (E) EMT‐associated protein expression was analyzed through a WB assay. *P < 0.05, **P < 0.01.

Subsequently, to test whether ESCCAL‐1 promotes ESCC progression via regulating miR‐590, KYSE150 and KYSE450 cells were subject to co‐transfection of si‐ESCCAL‐1 with miR‐590 inhibitor. As a result, miR‐590 silencing reversed the declined growth, migration, and invasion induced by ESCCAL‐1 knockdown (Figure 4A–D). In addition, the observation of protein expression indicated that co‐transfecting si‐ESCCAL‐1 with the miR‐590 inhibitor augmented N‐cadherin and Vimentin levels expression, whereas declined E‐cadherin level (Figure 4E). These data manifested that ESCCAL‐1 could interact with miR‐590 to affect ESCC cell phenotype.

### 3.5 LRP6 is the potential target of miR‐590

**FIGURE 5:**
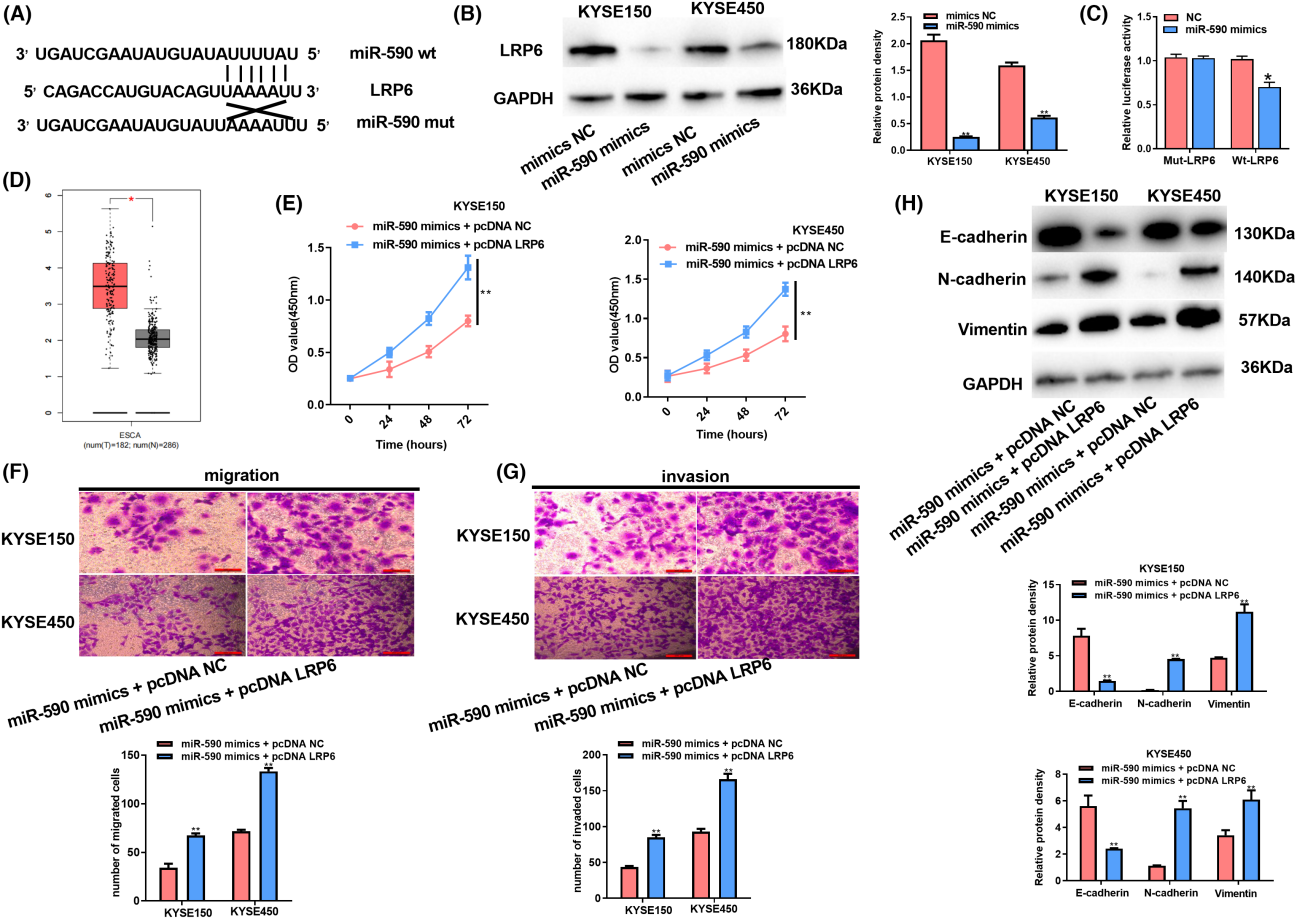
LRP6 is the potential target of miR‐590. (A) The bioinformatic analysis estimated LRP6 as miR‐590's putative target. (B) Western blot detected LRP6 protein levels within KYSE150/KYSE450 cells after miR‐590 mimics transfection. (C) Luciferase assay in cells after co‐transfection of MUT or WT LRP6 luciferase reporter plasmid and miR‐590 mimics. (D) LRP6 expression in ESCC tumor samples is predicted by the GEPIA website. (E) CCK‐8 assay evaluated cell proliferation abilities. (F) Cell migration was analyzed through the Transwell migration assay. (G) Cell invasion was analyzed through the Transwell invasion assay. (H) WB assay evaluated EMT‐related protein levels. *P < 0.05, **P < 0.01.

Subsequently, this work explored the molecular mechanism underlying miR‐590‐mediated attenuation of proliferation and migration in ESCC cells. Bioinformatics revealed that LRP6 3′‐UTR possessed the miR‐590 binding site, implying that LRP6 was the possible miR‐590 target (Figure 5A). As revealed by qRT‐PCR and WB assay, overexpression of miR‐590 remarkably down‐regulated LRP6 in KYSE150 and KYSE450 cells (Figure 5B–C). To further verify the direct binding effect, we generated pmirGLO‐LRP6‐wt and pmirGLO‐LRP6‐mut luciferase reporter constructs. As a result, luciferase activities decreased in HEK‐293 T subject to co‐transfection of miR‐590 mimic with pmirGLO‐LRP6‐wt relative to co‐transfection of pmirGLO‐LRP6‐mut with miR‐590 mimic (Figure 5D). Then we sought to analyze LRP6 expression in ESCC, and the GEPIA database displayed that it was up‐regulated (Figure 5E). Afterward, we were curious to examine whether miR‐590 inhibited the malignant phenotype of ESCC by targeting LRP6, rescue experiments with overexpression of LRP6 while transfection miR‐590 mimics were performed. The results showed that LRP6 over‐expression could reverse miR‐590's inhibition of cell growth, invasion, and migration (Figure 5E–G). Besides, LRP6 over‐expression reversed Vimentin, N‐cadherin, and E‐cadherin expression resulting from miR‐590 up‐regulation (Figure 5H). Together, these results provided that miR‐590 could inhibit cellular malignancy by targeting LRP6 in ESCC.

### 3.6 ESCCAL‐1 promotes growth, migration, and invasion of ESCC cells via miR‐590/LRP6/ axis in vitro

**FIGURE 6:**
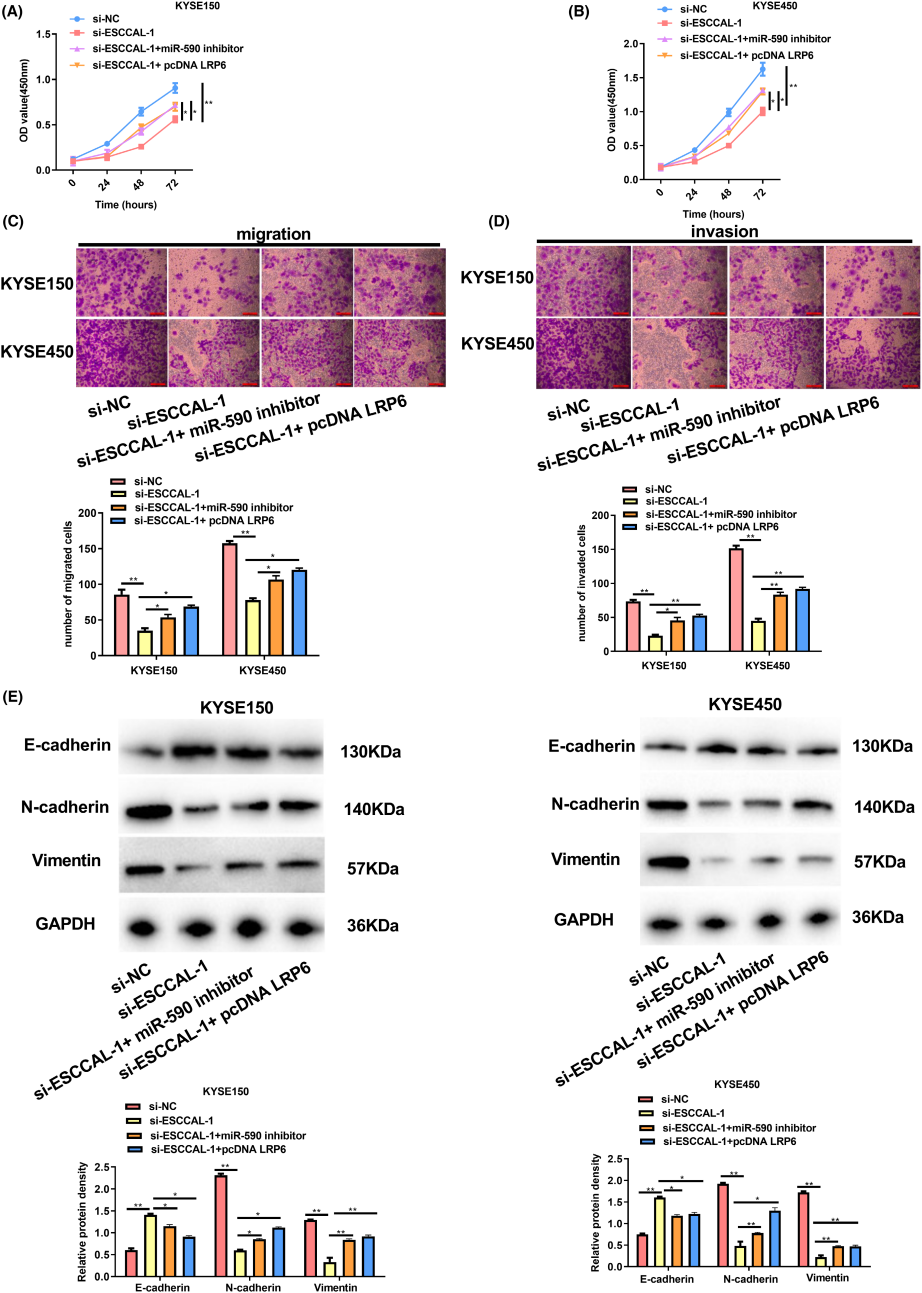
ESCCAL‐1 promotes ESCC cell growth, invasion, and metastasis by miR‐590/LRP6/ axis. si‐ESCCAL‐1 and miR‐590 inhibitor (or pcDNA LRP6) were co‐transfected into cells. (A) CCK‐8 assay evaluated KYSE150 cell growth. (B) CCK‐8 assay evaluated KYSE450 cell growth. (C) Cell migration was analyzed through the Transwell migration assay. (D) Cell invasion was analyzed through the Transwell invasion assay. (E) WB assay evaluated EMT‐related protein expression. *P < 0.05;**P < 0.01.

We hypothesized that ESCCAL‐1 facilitated growth, metastasis, and invasion of ESCC cells via the miR‐590/LRP6 axis based on the above data,. To fully understand the consequences of the ESCCAL‐1‐miR‐590‐LRP6 interactions in ESCC tumorigenesis, ESCCAL‐1 was knocked down, or LRP6 (miR‐590) was over‐expressed in KYSE150 and KYSE450 cells. The results showed that cell growth, invasion, and metastasis abilities were reduced within cells subject to si‐ESCCAL‐1 transfection. In contrast, the abilities were partially recovered when cells were co‐transfection with miR‐590 inhibitor or simultaneously overexpressed LRP6, indicating that ESCCAL‐1 regulated the miR‐590/ LRP6 axis to promote ESCC progression in vitro (Figure 6A–D). In addition, LRP6 over‐expression or miR‐590 inhibitor partly reversed the change of EMT‐related proteins expression induced by ESCCAL‐1 knockdown (Figure 6E). Collectively, the findings demonstrated that ESCCAL‐1/miR‐590/LRP6 interacts to regulate ESCC cellular malignancy.

### 3.7 ESCCAL‐1 promotes ESCC tumor growth in vivo

**FIGURE 7:**
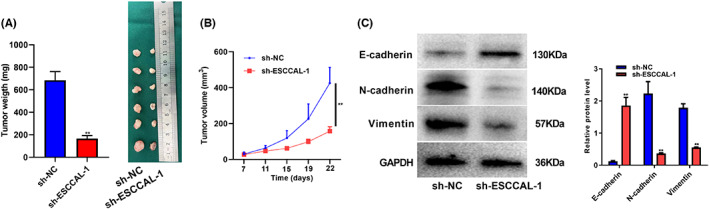
ESCCAL‐1 promotes ESCC tumor growth in vivo. A nude mouse model of esophageal cancer subcutaneous xenografts was constructed. ESCCAL‐1 silenced esophageal cancer cells (sh‐ESCCAL‐1, *n* = 6) and negative control group KYSE150 esophageal cancer cells (sh‐NC, *n* = 6) were inoculated, respectively. (A) The Tumor weight of the sh‐ESCCAL‐1 group was significantly lower than that of the sh‐NC group. (B) The tumor proliferation rate of nude mice was slowed down after ESCCAL‐1 silencing. (C) EMT‐related protein expressions were determined. **P < 0.01.

The above results confirmed the effect of ESCCAL‐1 on the malignant phenotype of esophageal cancer cells in vitro. To verify the above results in vitro, a nude mouse model of esophageal cancer subcutaneous xenografts was constructed in this study. The xenograft model was successfully constructed when a noticeable mass can be palpated subcutaneously in nude mice. After the model was successfully constructed, the long and short diameters of the transplanted tumor were measured, and the change in the growing volume of the transplanted tumor was calculated. The mice were sacrificed after 22 days of feeding, and the transplanted tumors were weighed. The results confirmed that the tumor weight of the sh‐ESCCAL‐1 group was significantly lower than that of the sh‐NC group (Figure 7A). The results of tumor volume also confirmed that the tumor proliferation rate of nude mice was slowed down after ESCCAL‐1 silencing (Figure 7B). Meanwhile, the expression of EMT‐related proteins was also determined. The results showed that N‐cadherin and Vimentin protein levels were decreased, but E‐cadherin protein levels were increased in the sh‐ESCCAL‐1 group (Figure 7C). Collectively, these data suggest that ESCCAL‐1 promotes ESCC tumor growth in vivo.

## 4 DISCUSSION

A large body of evidence shows that lncRNAs often are altered within numerous human disorders like epilepsy, Alzheimer's disease, male infertility, cardiovascular disease, and cancer.^24^, ^25^, ^26^, ^27^, ^28^, ^29^, ^30^ LncRNAs play roles of tumor suppressor genes or oncogenes, and promote or inhibit proliferation, invasion, and metastasis in many types of cancers, including ESCC. For example, activation of CCAT1 by H3K27 acetylation affected ESCC proliferation and migration through SPRY4 and HOXB13 exhibited distinct regulatory mechanisms in the nucleus and cytoplasm, respectively.^31^ SNHG1, a well‐known oncogene, was recently highlighted to affect cell Epithelial‐Mesenchymal Transition (EMT), growth, and invasion by modulating the Notch pathway.^32^ ESCCAL‐1, also called CASC9, was proved to facilitate the malignancy of CRC, EC, BC, lung cancer, glioma, ovarian cancer, and gastric cancer (GC).^33^, ^34^, ^35^, ^36^, ^37^, ^38^, ^39^, ^40^, ^41^ Wu et al. showed that CASC9 contributed to carcinogenesis through the negative regulation of PDCD4 level via recruitment of EZH2 and subsequently affecting H3K27me3 level.^34^ Likewise, it was reported that CASC9 expression was high in ESCC and its knockdown significantly suppressed ESCC cell invasion and metastasis in vitro. Based on this work, silencing ESCCAL‐1 restrained xenograft proliferation in the mouse model of ESCC xenograft via inactivating Src while activating the p38α pathway.^6^ Here, we aimed to unravel the mechanism by which ESCCAL‐1 mediated ESCC development. After extending the previous findings, we discovered ESCCAL‐1 up‐regulation within human ESCC cells and tissues. Also, ESCCAL‐1 knockdown significantly decreased cell proliferation, invasion, and migration, elucidating that ESCCAL‐1 functions as an oncogene in ESCC.

Recently, a new regulatory mechanism has emerged for lncRNAs to interact with miRNAs in cancer. In this study, bioinformatics predicted miR‐590 as a potential target of ESCCAL‐1, which was confirmed by luciferase assay. Furthermore, silencing ESCCAL‐1 increased miR‐590 expression in KYSE150 and KYSE450 cells. Here, miR‐590 showed decrease within ESCC. while its over‐expression suppressed ESCC cell growth, invasion, and metastasis. Besides, miR‐590 showed a negative correlation with ESCCAL‐1 within ESCC tissues. Based on the above findings, ESCCAL‐1 could interact with miR‐590 and restrict its expression, and miR‐590 plays a tumor‐suppressing role within ESCC.

We predicted and confirmed the miR‐590 binding to LRP6 3′‐UTR. In addition, we observed increased LRP6 expression in ESCC, and LRP6 over‐expression could reverse miR‐590's inhibition of cell growth, invasion, and metastasis. More importantly, LRP6 over‐expression partly reversed EMT‐related protein expression resulting from miR‐590 over‐expression. Collectively, miR‐590 could inhibit cellular malignancy and decrease oncogenic functions by targeting LRP6.

Given that ESCCAL‐1 could interact with miR‐590 and suppress its expression, we speculated that a regulatory network of the ESCCAL‐1/miR‐590/LRP6 signal pathway controls the growth and metastatic potentials of ESCC cells. By performing a series of experiments, we revealed that over‐expression of LRP6 or inhibition of miR‐590 partly reversed the inhibition roles of ESCCAL‐1 silencing in cell proliferation, invasion, and metastasis; more importantly, a similar trend also was observed in EMT related protein expression; strongly demonstrating that ESCCAL‐1 promoted ESCC progression mediated modulation miR‐590/LRP6. Thus, collectively, it is likely to conclude that decreased ESCC cell growth, invasion, and metastasis observed by ESCCAL‐1 knockdown were partly due to increased miR‐590 expression and subsequent inhibition of LRP6.

In summary, we manifested that a novel lncRNA ESCCAL‐1 promoted ESCC cell malignant behavior through a regulatory network of the ESCCAL‐1/miR‐590/LRP6 axis. Targeting this regulatory network is a promising and effective anti‐ESCC treatment.

## AUTHOR CONTRIBUTION

Qinghe Xing and Wei Cao designed the experiments. Hongya Guan, Pengju Lv, Pengli Han and Jia Liu performed the periments. Lijuan Zhou undertook the statistical analysis. Hongya Guan drafted the manuscript. Ming Yan and Wei Wu helped revised the manuscript.

## CONFLICT OF INTEREST

No conflict of interest.

## ETHICS STATEMENT

The Research Ethics Committee of Zhengzhou Central Hospital Affiliated to Zhengzhou University (approval no. 201910) approved the research. The in vivo tumor xenograft experiments were implemented under the permission of the Laboratory Animal Platform of Zhengzhou University Academy of Medical Sciences.

## Data Availability

The datasets used and/or analyzed during the present study are available from the corresponding author upon reasonable request.
